# Clinical evaluation of a computer-assisted decision support and documentation system for the primary care of polytrauma patients

**DOI:** 10.3389/fdgth.2026.1776960

**Published:** 2026-06-26

**Authors:** Lisa Bruckelt, Juliane Neumann, Annette Keß, Notker Blankenburg, Thomas Neumuth, Christian Kleber, Georg Osterhoff

**Affiliations:** 1 Department of Orthopaedics, Trauma and Plastic Surgery, University Hospital Leipzig, Leipzig, Germany; 2 Innovation Center Computer Assisted Surgery (ICCAS), Medical Faculty, University of Leipzig, Leipzig, Germany; 3 Department of Trauma Surgery and Orthopaedics, BG Klinikum Unfallkrankenhaus Berlin, Berlin, Germany; 4 Centrum für Muskuloskelettale Chirurgie, Charité-Universitätsmedizin Berlin, Berlin, Germany

**Keywords:** clinical decision support systems, documentation, guideline adherence, polytrauma, trauma, workload

## Abstract

**Background:**

Polytrauma management demands rapid, high-stakes decisions under severe time pressure, increasing the risk of error and deviation from evidence-based protocols. Computer-assisted decision support systems (CDSS) may counter these risks by improving adherence to guidelines and reducing cognitive strain. We developed TraumaFlow, a CDSS integrating the German S3 guideline and ATLS® principles, and evaluated its impact in real-world trauma care.

**Methods:**

In a prospective study at a level 1 trauma center, 30 shock room cases were managed with TraumaFlow alongside the standard paper protocol and compared both with the corresponding paper documentation and with 30 conventionally documented cases from a historical cohort. System-generated recommendations were tracked for implementation. Usability was assessed by questionnaire, and workload measured with the NASA Raw Task Load Index (NASA-RTLX).

**Results:**

Eighteen residents (mean age 31 ± 4 years, 56% female) participated in 30 polytrauma cases. Documentation completeness was significantly higher with TraumaFlow compared to paper (20.5/25 items (82%) vs. 18.4/25 items (74%), *p* = 0.002). Of 74 clinical prompts, 37% triggered clinically relevant actions that might otherwise have been missed. Workload did not differ significantly between TraumaFlow-supported and conventional cases (35.0 ± 12.4 vs. 34.7 ± 15.3). Participants rated usability positively and reported improved confidence.

**Conclusion:**

TraumaFlow enhanced documentation quality and supported guideline-based management in acute polytrauma care. Although no measurable reduction in workload was observed, the system improved structure, reduced risk of omission, and was well accepted by clinicians. These findings highlight the potential of CDSS to strengthen trauma team performance and standardize complex emergency care.

**Clinical Trial Registration:**

https://drks.de/register/de/trial/DRKS00034201, Registration No. DRKS00034201.

## Introduction

The management of polytrauma patients is one of the most time-critical and stressful challenges in acute surgical care. The need for rapid decision-making in high-pressure environments increases the risk of treatment errors, potentially compromising patient outcomes (1). To address these challenges, the integration of computer-assisted decision support systems (CDSS) has been proposed as a promising tool to support medical teams. Such systems aim not only to enhance adherence to clinical guidelines, thus improving clinical results, but also to reduce cognitive load and stress among healthcare professionals (1, 2).

Accurate and complete documentation is relevant for quality management and of particular importance in Germany, where the recording of trauma patients in the TraumaRegister DGU® is mandatory, leading to a substantial documentation workload for clinical staff. In the context of automating and streamlining these processes, the authors developed “TraumaFlow” (3, 4), a guideline-based CDSS tailored for polytrauma patient care drawing on the 2018 German S3 guideline for the treatment of severely injured patients (5) and ATLS® principles (6), TraumaFlow collects and processes patient data in real time, provides structured decision support, and facilitates documentation throughout the treatment process. Previous *in-vitro* evaluations of TraumaFlow demonstrated improved adherence to clinical guidelines and reduced stress levels in fast-paced trauma care scenarios (3, 4). In contrast to many previously described CDSS approaches in trauma care, which primarily provided more general forms of decision support and lacked real-time, context-sensitive, and patient-specific decision support (1, 7), TraumaFlow integrates these elements within a continuous workflow-based system while at the same time providing documentation of the shock room treatment. However, evidence regarding the clinical implementation and real-world impact of such workflow-integrated CDSS in trauma care remains limited. Therefore, the aim of this study was to evaluate the clinical application of TraumaFlow in a real-life trauma care setting. Specifically, we investigated whether the system improves compliance with clinical guidelines and the completeness and accuracy of documentation, while also reducing perceived stress among trauma team members and achieving high user satisfaction.

## Methods

### Participants

All physicians at the Department of Orthopedics, Trauma Surgery, and Plastic Surgery (OUP) involved in emergency room management of severely injured patients were trained via video instruction in the use of TraumaFlow prior to the study. The department is part of a Level I trauma center.

To evaluate the system's effectiveness, the study was divided into two comparative parts based on the documentation method used during shock room care.

In the first part, emergency room management and documentation were performed by a physician using the digital support system TraumaFlow. Subsequently, a conventional paper-based protocol was completed in accordance with standard clinical procedures. This part included a total of 30 polytrauma patients treated by 18 different residents.

The second part of the study (*n* = 30) consisted of trauma cases managed and documented exclusively using the conventional paper-based approach without the aid of TraumaFlow. This group represented a retrospective historical cohort comprising consecutive trauma treatments performed immediately prior to the prospective implementation of TraumaFlow.

The resulting documentation from both parts was later compared regarding quality and completeness. Additionally, a further cohort (*n* = 10), which exclusively completed the paper-based protocol, was prospectively recorded for workload assessment. The perceived workload in this group was subsequently compared with that of the TraumaFlow-assisted group.

**Figure 1:**
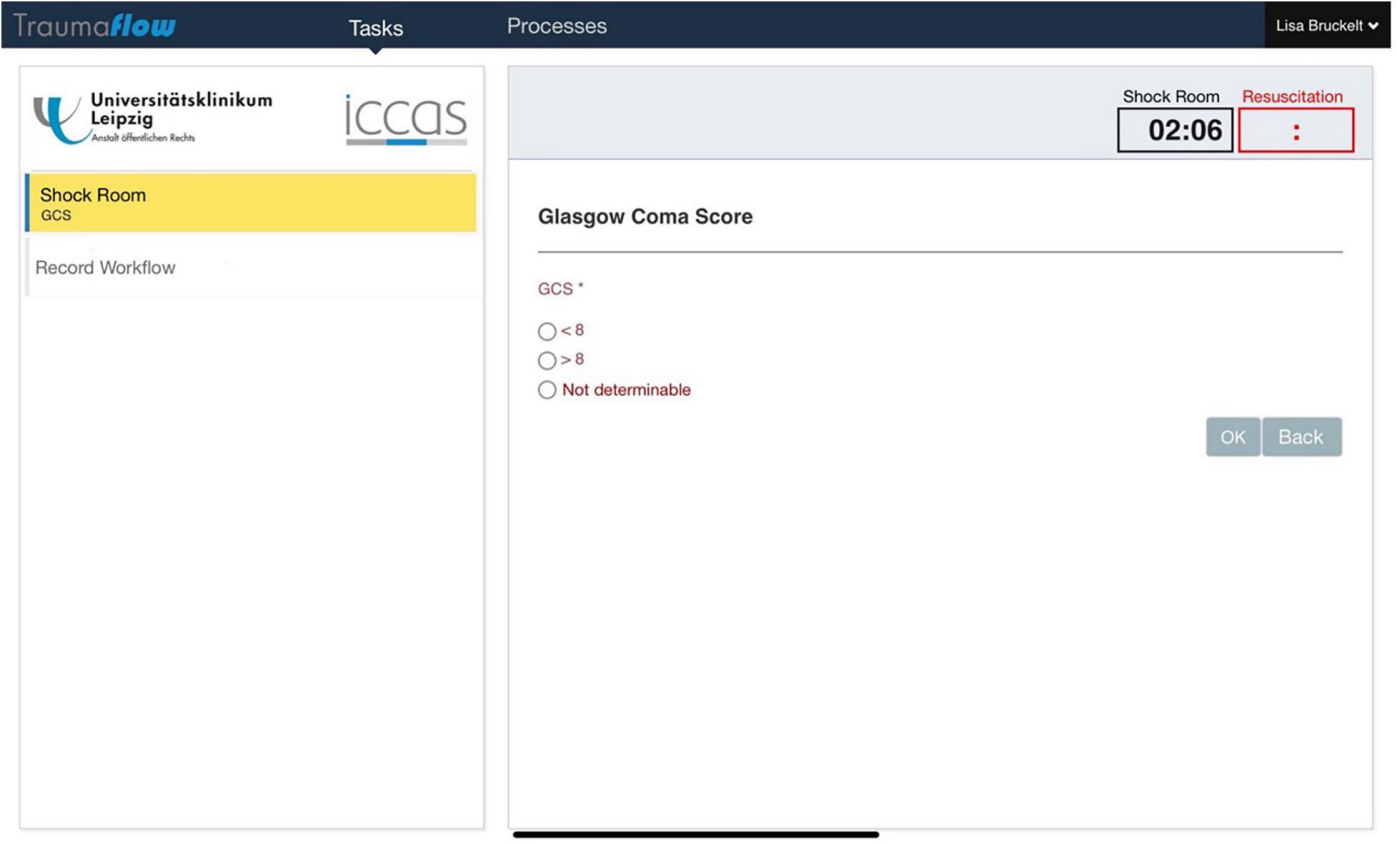
Screenshot of the TraumaFlow user interface.

Personal data collected on the participants included age, gender, year of residency and the number of times TraumaFlow was used. All participants used the same version of TraumaFlow, a CDSS operated on a tablet computer (Figure 1).

Prospective data collection was conducted between 16 December 2024 and 23 May 2025. Data for the historical cohort were retrospectively collected from trauma cases treated between 24 November 2024 and 13 December 2024.

The protocol of this study was approved by the local ethics committee (reference 194/24-ek).

### Comparison of the documentation methods

TraumaFlow guides the clinical workflow through structured, real-time queries and simultaneously generates a largely pre-filled protocol based on the entered data, that can be edited afterwards. Additionally, the system provides real-time, guideline-based recommendations to support clinical decision-making during shock room management (Figure 1). The paper-based protocol, in contrast, has predefined fields, primarily focusing on patient-related data and key aspects of shock room procedures, that have to be manually completed. No structured guidance or prompts are provided during documentation.

### Polytrauma cases

The study included consecutive cases that triggered trauma team activation and that sustained at least one traumatic injury.

In the first part, in which TraumaFlow was used, prehospital triage levels classified 20 cases as yellow alert and 8 as red alert; 2 patients were pediatric cases. Fourteen patients were secondarily transferred from other hospitals. The distribution of primary diagnoses was as follows: 12 patients presented with acquired brain injury, 6 with thoracic trauma, 4 with stab or cut wounds, 4 with spinal injuries, 4 with extremity fractures, 3 with organ contusions, 1 with pelvic fracture, and 1 patient was diagnosed with septic shock. Regarding injury severity as a determinant of case complexity and workload, the cohort included patients with varying ISS levels (10 patients ISS < 9, 6 patients ISS 9–15, and 5 patients ISS ≥ 16). For the remaining 9 patients, insufficient information was available to reliably calculate the ISS.

In the second part (*n* = 30), management and documentation were carried out according to the hospital's standard procedures without the use of TraumaFlow prior to the start of the prospective study.

Of these patients, 9 were secondarily transferred from other hospitals. Prehospital triage classified 15 patients as yellow alert and 9 as red alert; 4 patients were pediatric cases. In 2 cases, the available information was insufficient for classification. The distribution of primary diagnoses in this group included 16 cases of acquired brain injury, 3 extremity fractures, 3 stab or cut wounds, 2 spinal injuries, 1 organ contusion, and 1 case of pelvic infection. Fourteen patients had an ISS < 9, while 5 patients had an ISS ≥ 9. For 11 patients, insufficient clinical information was available to calculate the ISS reliably.

### Assessment of impact on compliance with clinical guidelines

If feasible on short notice, the first author attended the shock room following notification of an incoming polytrauma case to observe the interaction between the physician using TraumaFlow and the remaining trauma team. During these treatments, the observer also documented clinical recommendations generated by TraumaFlow that influenced patient management. While many recommendations were implemented implicitly during ongoing care, some addressed actions that had not yet been performed and therefore required active consideration by the team. These recommendations were either explicitly acknowledged and subsequently overlooked, overruled, or actively incorporated into the treatment process. Such instances were assessed, categorized, and documented in their respective clinical context by the first author during direct observation.

### Assessment of perceived workload and usability

After each polytrauma treatment, the participants were asked to complete questionnaires on perceived workload during the cases. For this purpose, the NASA Raw Task Load Index (NASA-RTLX) was utilized (8). The NASA-RTLX is a multidimensional score with six subscales: mental demand, physical demand, temporal demand, frustration, effort, and performance. After the completion of each scenario, the performed tasks were rated by the participant on a visual analog scale (0–100). In addition, the overall workload is calculated by a combination of all six dimensions. The questionnaire used represents a validated instrument for the assessment of user experience of interactive products (9). Furthermore, the usability of the digital interface was assessed through a combination of closed- and open-ended questions completed by the participants, using a questionnaire developed specifically for this study (see Supplementary File 1). The questionnaire had previously been applied and tested in the preceding *in-vitro* evaluation of TraumaFlow (3, 4). It included items addressing perceived learning effects, decision support, usefulness of the recommended actions, and usability of the user interface. Participants rated their agreement with the statements using a Likert-type scale and were additionally invited to provide free-text feedback. As the questionnaire was developed specifically for the TraumaFlow evaluation, no formal psychometric validation has been performed to date.

### Assessment of documentation quality

To evaluate the quality of documentation, a comparative analysis was conducted between protocols completed with and without the use of TraumaFlow. Specific sections (*n* = 25), identical for both documentation modalities, including vital signs, diagnostic procedures, and therapeutic interventions, were assessed individually for completeness. Blank fields were considered incomplete, as information on the presence of the respective variable was missing. Additionally, the overall completeness of each protocol was analyzed. To evaluate consistency, corresponding entries in the digital TraumaFlow documentation and the conventional paper-based protocols were compared for agreement. This approach allowed for a structured assessment of both the thoroughness and internal consistency of both documentation practices.

### Statistical analysis

Statistical analyses were performed using SPSS 27.0 (SPSS Inc., Chicago, IL, USA). Data were summarized as mean with standard deviation (SD). Where applicable, nominal variables crosstabs were associated using Chi-Square or Fisher's Exact tests. Student's *t*-test was used to detect differences in means of normally distributed continuous data. Paired tests were used for comparison of the two scenarios among the same participant. To achieve a power of 80% and an alpha-error of 0.05, we aimed for a sample size of *n* = 29 polytrauma cases. The level of significance was defined as *p* < 0.05. Assuming an effect of at least 2 performance points (SD 2) when using TraumaFlow and aiming for a power of at least 0.80, the minimum sample size was calculated to be *n* = 10.

## Results

**Figure 2:**
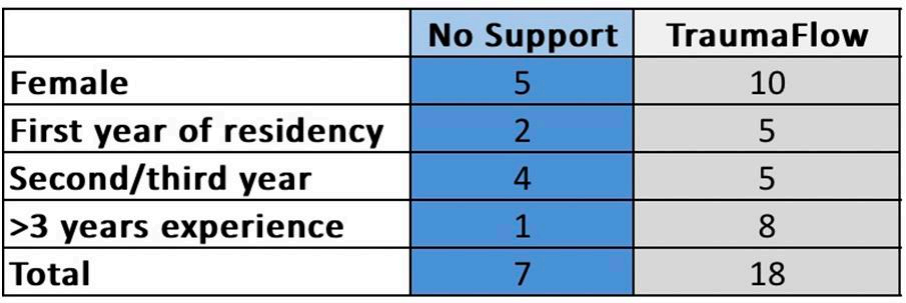
Participant characteristics.

For the prospective arm of the study using TraumaFlow, a total of 18 physicians (mean 31 ± 4 years, Figure 2) participated. Ten participants used TraumaFlow only once during the study period, while in 20 additional trauma cases, the system was used multiple times by the remaining 8 residents.

A separate prospective cohort using the paper-based protocol (*n* = 10) was included for workload assessment. The group consisted of 7 physicians (mean 31 ± 4 years, Figure 2), of whom 3 provided repeated workload assessments.

### Assessment of compliance with clinical guidelines

In 30 observed trauma resuscitations, TraumaFlow influenced or contradicted a total of 74 clinical actions. Since the system operates in parallel with real-time clinical care, many of its suggestions were implemented by the trauma team without requiring explicit communication or active relay by the TraumaFlow user.

The performed recommendations were categorized according to the ABCDE framework:

A (Airway): 16%, B (Breathing): 0%, C (Circulation): 39%, D (Disability): 3% and E (Exposure/Environment): 42%.

Out of all the 74 system recommendations, 47% were neither implemented nor discussed. The most frequent omission was the failure to administer tranexamic acid (TXA) in 10 cases with potential indications.

Sixteen percent of the system recommendations were considered inappropriate for the specific scenarios, such as cervical spine immobilization without evidence of cervical injury (*n* = 8) or suggesting a tetanus booster in the absence of open wounds (*n* = 3).

Notably, 37% of recommendations were actively implemented and may otherwise have been overlooked. The most frequent were establishing two intravenous lines (*n* = 6), verifying tetanus immunization status (*n* = 6), and performing a full physical examination (*n* = 4).

### Documentation quality

**Figure 3:**
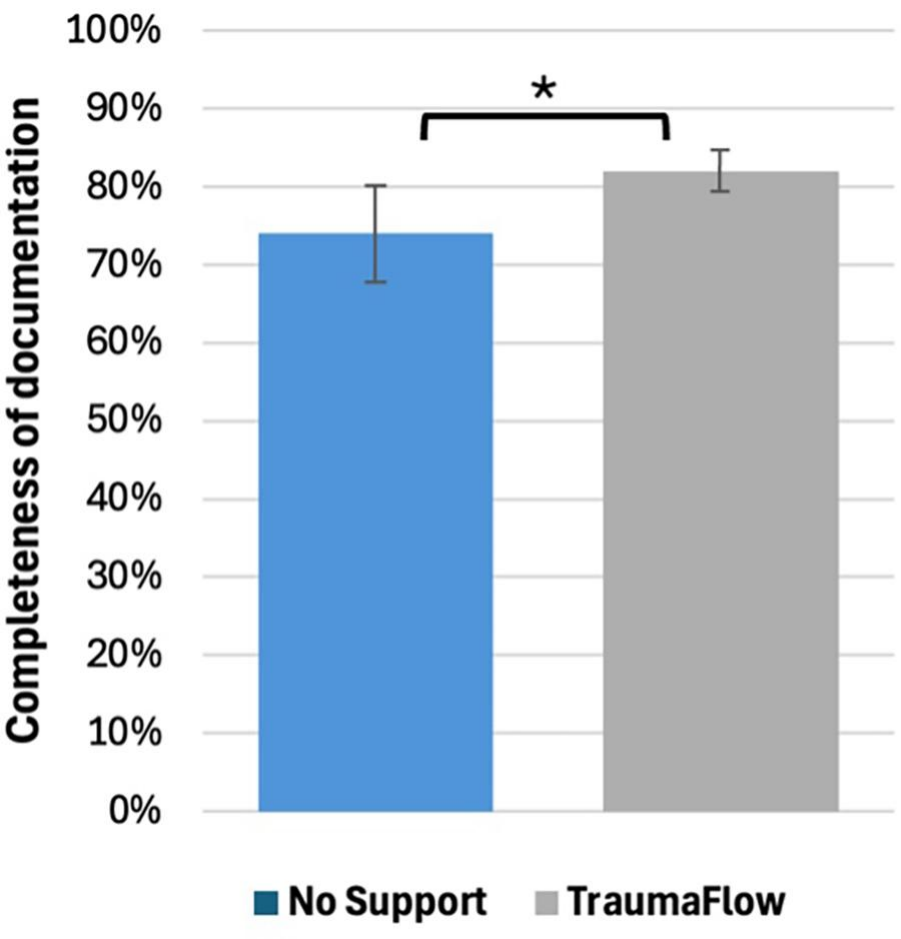
Completeness of documentation (in %), * denotes statistical significance.

Analysis of the 25 defined protocol items revealed that, on average, TraumaFlow-assisted protocols contained mean 20.5 (SD 1.54) fully completed protocol items and 1.4 entirely empty ones. In contrast, the same protocols completed without the help of TraumaFlow had an average of 18.4 (SD 3.04) fully completed (18.4/25 items (74%) vs. 20.5/25 (82%), *p* = 0.002, see Figure 3) and 3.6 completely empty items (3.6/25 items (14%) vs. 1.4/25 items (6%), *p* < 0.001). Among the 30 protocols analyzed, 19 had a higher number of fully completed fields when supported by TraumaFlow, while 3 contained more entirely blank fields than their corresponding parts. In 6 cases, the number of fully completed and empty categories remained identical regardless of TraumaFlow use.

A notable difference was also observed in the number of completed checkbox items within the protocols, with participants completing on average 8.4 of the nine possible items when using TraumaFlow, compared to 7.7 without TraumaFlow (8.4/9 items (93%) vs. 7.7/9 items (86%), *p* = 0.02).

Content-related discrepancies were identified in 17 instances across the dataset. The most frequent inconsistency was in the Injury Severity Score (ISS), occurring in 5 cases.

Other discrepancies were related to documented volume administration, GCS scores and admission data, among other elements.

In an additional retrospective historical control group, where protocols had been completed exclusively without the use of TraumaFlow, a similar pattern was observed. Compared to the protocols completed by the help of TraumaFlow, the historical protocols showed significantly fewer fully completed (average 18.6/25 items (74%) vs. 20.5/25 items (82%), *p* < 0.001) and more entirely empty (average 3.7/25 items (15%) vs. 1.4/25 items (6%), *p* < 0.001) items.

A significant difference was also observed for the checkbox items, with participants completing on average only 7.1 of the possible options (8.4/9 items (93%) vs. 7.1/9 items (79%), *p* < 0.001), while participants in the TraumaFlow group reached an average of 8.4 items.

### Perceived workload and usability

Eighteen participants completed 30 shock room scenarios with TraumaFlow decision support, while seven participants in the control group completed 10 scenarios without such support. The subjective overall workload with TraumaFlow (*n* = 30) was rated as “high” in two instances, “somewhat high” in seventeen, “medium” in ten, and “low” in one. In the control group (*n* = 10), it was rated as “high” in two instances, “somewhat high” in four, and “medium” in four. No participant in either group rated the workload as “very high”.

**Figure 4:**
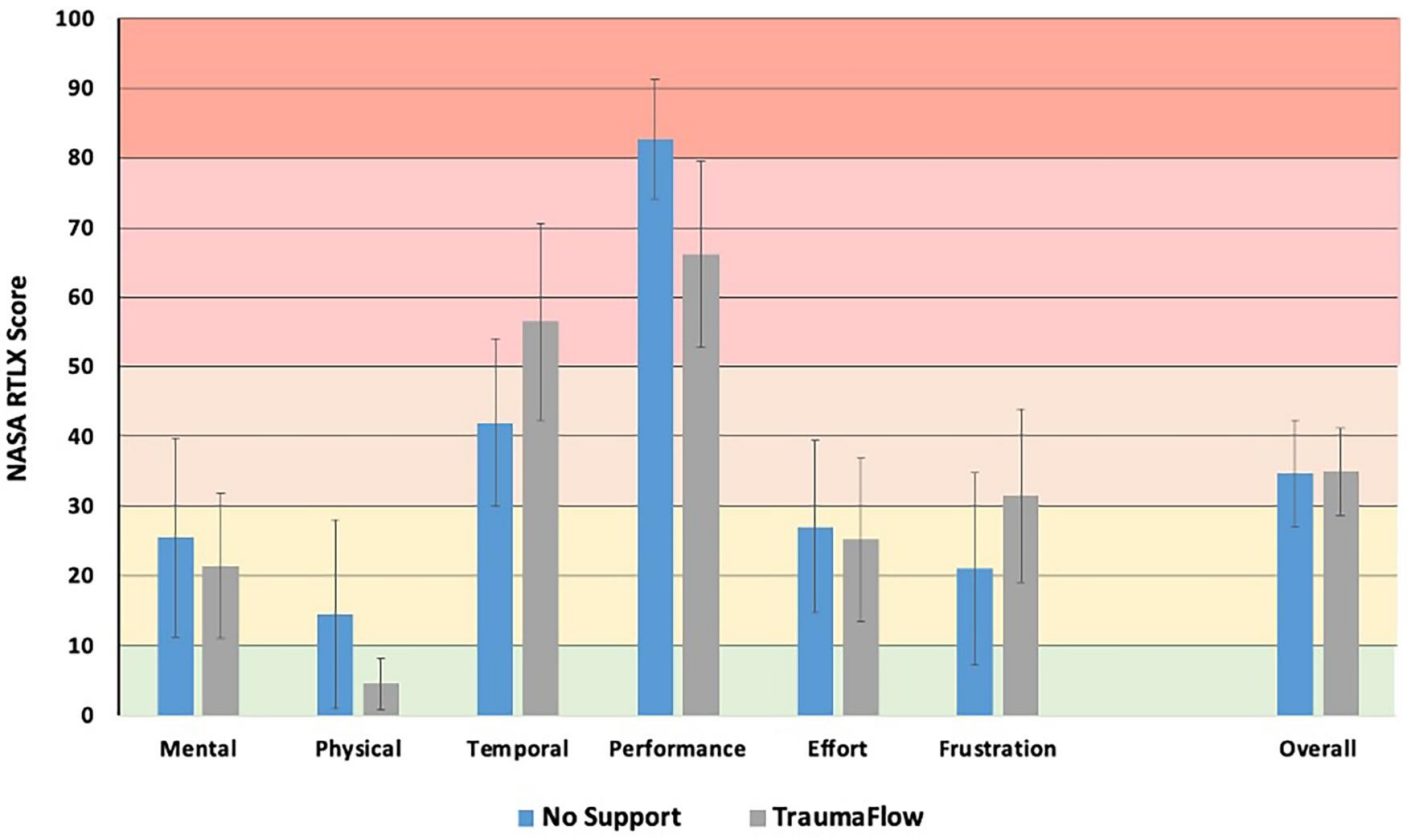
Stress as measured by NASA RTXL with and without computer assistance.

There was no significant difference in the NASA-RTLX overall score (Figure 4) between scenarios with TraumaFlow (35.0, SD 12.4) and without decision support (34.7, SD 15.3). While physical stress levels were lower with the CDSS (4.5, SD 7.4) compared to the control condition (14.5, SD 27.2), this difference did not reach statistical significance (*p* = 0.077). Similarly, no significant difference was found for self-reported mental stress (TraumaFlow: 21.4, SD 20.8; control group: 25.5, SD 28.6).

**Figure 5:**
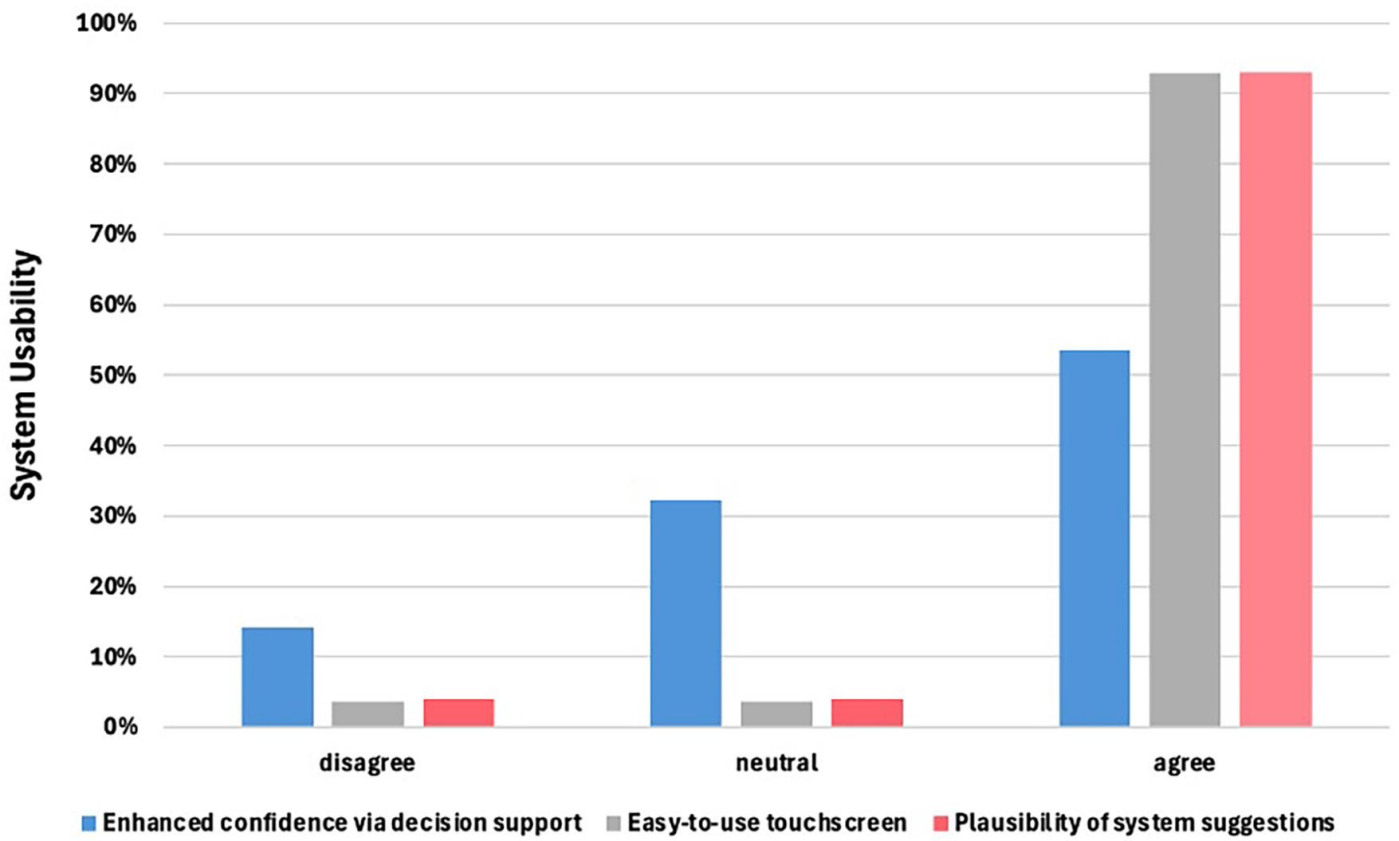
System usability (in %).

No objective improvement in performance or stress reduction was detected. Some participants found the system overly detailed, yet the majority rated the user interface positively, reported smooth touchscreen operation, and considered the suggestions plausible. Several expressed enhanced confidence with computer-based decision support (Figure 5).

## Discussion

In this study, we evaluated the clinical implementation of the computer-assisted decision support system TraumaFlow during real-life emergency management of polytrauma patients. Our results demonstrate that the system supports a structured, guideline-based approach and enhances documentation completeness, while no measurable reduction in subjective workload, as assessed by the NASA Task Load Index, was observed. TraumaFlow-supported cases showed a higher degree of protocol completeness compared to conventional paper-based documentation. The structured digital interface, combined with real-time guidance, likely contributed to this improvement. By providing context-sensitive recommendations and an integrated documentation workflow, the system may ultimately reduce bureaucratic workload, even though initial use requires additional time and adaptation by the clinical staff. One important limitation of this study is the comparison with a historical cohort, as the composition of the physicians was not entirely identical between groups and different patients were treated in each cohort. Since no trauma bay case is fully comparable to another, achieving complete cohort equivalence and perfect comparability is challenging, particularly in a study with a relatively small sample size such as this one.

Across both physical and mental workload dimensions, average scores were lower in the TraumaFlow group. Despite this trend, no statistically significant reduction was observed. Some physicians noted that the detailed documentation requirements and frequent prompts increased perceived effort, potentially counteracting stress-reducing effects. This suggests that while TraumaFlow enhances process quality and documentation accuracy, its immediate influence on stress levels may be limited—particularly for more experienced clinicians, who already rely on established routines. Future studies may investigate factors influencing mental and physical workload, as measured by the NASA Task Load Index.

However, the emergency room is a high-pressure environment (10) and most participants in both groups reported their stress levels as “medium” or higher, with only a single respondent rating stress as “low”. More frequent use of the system as well as structured training prior to implementation could help users interact more efficiently with the interface and may enhance its stress-relieving potential.

Several other limitations must be acknowledged. The sample size was relatively small and limited to a single institution. TraumaFlow use differed between participants, with some physicians using it only once, which may have restricted familiarity and efficiency. Consequently, stress-reducing effects may not yet be visible after initial exposure. Furthermore, user experience and perceived workload may have been affected by differences in participants' clinical experience and familiarity with trauma care, as well as by variations in the severity and complexity of the treated trauma cases. In addition, potential bias may have been introduced by interindividual differences in documentation styles, particularly in the paper-based group, which could have affected the consistency and comparability of the recorded data.

Despite these limitations, the majority of participants evaluated the user interface positively, describing the touchscreen operation as smooth and the clinical suggestions as plausible. Several reported enhanced confidence in their management decisions when using the system, highlighting an important psychological benefit: even in the absence of measurable stress reduction, structured support can reinforce correct clinical behavior (11). These findings align with previous research demonstrating that digital decision aids improve guideline adherence as well as increased confidence (2), resulting in better outcomes (1, 12, 13). In contrast to the earlier *in-vitro* studies on TraumaFlow (3, 4), the present investigation assessed its performance in real-world trauma care, involving patients with heterogeneous injury patterns and varying triage levels. TraumaFlow was able to provide context-sensitive, real-time recommendations tailored to these diverse clinical scenarios. A substantial proportion of these recommendations were implemented, including essential but sometimes overlooked measures such as establishing two IV lines, performing comprehensive physical examinations, and verifying tetanus immunization. These results highlight the potential of such systems to improve adherence to guideline-based actions in dynamic trauma situations, which may potentially contribute to improved patient care, as previously discussed (1, 12, 13).

Nevertheless, the study was not designed to assess patient-centered outcomes, leaving open the question whether improved documentation and adherence will ultimately translate into better clinical results. Patient outcomes in this context could be assessed by comparing clinically relevant endpoints between groups, including in-hospital mortality, complication rates, length of stay in the intensive care unit and overall hospital stay, as well as time to critical interventions such as imaging, surgery, or transfusion.

Future development should aim to better balance the advantages of comprehensive documentation with the additional cognitive load. Automated data import from monitoring devices or imaging systems could streamline workflows and further reduce effort. Successful integration of systems like TraumaFlow also requires sufficient digital infrastructure, such as tablets and wireless networks, and will initially involve additional integration burden (14). However, long-term benefits may include error reduction, fewer complications, and more efficient use of resources. Hospitals with lower case volumes and less experienced trauma leaders may particularly benefit from such systems, as they provide standardized guidance and compensate for limited routine exposure. Nonetheless, it remains to be clarified to what extent clinicians in high-stress environments are willing to rely on CDSS for decision-making support, particularly in situations requiring a balance between guideline adherence and individualized clinical judgment.

Expanding TraumaFlow with artificial intelligence (AI) modules represents a promising next step. AI-assisted systems have the potential to enhance real-time decision-making and may positively impact patient outcomes (15). However, before such technologies can be widely implemented in clinical care, several challenges must be addressed, including technological limitations and concerns regarding the delegation of clinical decisions to AI systems (16). As AI-based extensions were not evaluated in the present study, their potential benefits and limitations remain beyond its scope.

TraumaFlow demonstrates that a computer-assisted decision support system with a user-friendly interface may reduce documentation burden over time and represents a valuable tool for structured and standardized trauma management.

## Conclusion

TraumaFlow enhanced documentation quality and supported guideline-based management in acute polytrauma care. Although no measurable reduction in workload was observed, the system improved structure, reduced risk of omission, and was well accepted by clinicians. These findings highlight the potential of CDSS to strengthen trauma team performance and standardize complex emergency care.

## Data Availability

The raw data supporting the conclusions of this article will be made available by the authors, without undue reservation.
