# Supplementary material for: Clinical evaluation of a computer-assisted decision support and documentation system for the primary care of polytrauma patients
Source: Front Digit Health. 2026 Jun 26;8:1776960. doi: 10.3389/fdgth.2026.1776960 (PMC13350439; doi:10.3389/fdgth.2026.1776960)
Supplement: Supplementary file 1 [file Datasheet1.pdf]

Universitätsklinikum Leipzig
Medizin ist unsere Berufung.

Number of Participant.: ______________________

Overall evaluation of the system

	Disagree completely	Disagree	Neutral	Agree	Agree completely
Computer-based decision support made me feel more confident in my approach.	○	○	○	○	○
The system reacted as expected.	○	○	○	○	○
The system can be used to specifically train polytrauma management.	○	○	○	○	○
The subjective training effect with system support was higher than without it.	○	○	○	○	○
The system helps to make the right decisions in a shock room situation.	○	○	○	○	○
The system can be operated easily via touchscreen.	○	○	○	○	○
The system's suggestions were plausible.	○	○	○	○	○

Optional questions

1. Do you think there is room for improvement?

2. Which features did you like or dislike about TraumaFlow?

3. Additional comments

Thank you for participating!
